# N-doped carbon dots from phenol derivatives for excellent colour rendering WLEDs†

**DOI:** 10.1039/c7ra12522d

**Published:** 2018-01-26

**Authors:** Qian Liu, Danting Li, Zhifeng Zhu, Shimeng Yu, Yan Zhang, Dabin Yu, Yang Jiang

**Affiliations:** School of Materials Science and Engineering, Hefei University of Technology Hefei 230009 P. R. China apjiang@hfut.edu.cn; State Key Laboratory of Pulsed Power Laser Technology, National University of Defense Technology Hefei 230037 P. R. China dbyu@ustc.edu

## Abstract

To achieve competitive fluorescence carbon dots (CDs), studies on regulating fluorescence of CDs under controlled, comparable conditions are in great demand. Herein, by changing the functional groups and nitrogenous existence forms in the precursors, three efficient yellow-green emissive N-doped CDs which have the same fluorescence peak wavelength but different photoluminescence quantum yields were realized through a facile hydrothermal method. The as-prepared CDs exhibit not only excited-independent emissions but also similar surface states. The best-performing CDs among the three products exhibits photoluminescence quantum yields of up to 24.4% in water and 53.3% in ethanol, abundant surface functional groups and its high N-doping degree would be the reason for its excellent performances. By washing and reduction processes, the emission evolution of the CDs was studied linking the changes of surface states. The fluorescence can certainly be attributed to the surface of the carbon dots, and the surface states control the photoluminescence features. Serving as a yellow-green colour conversion layer, the best CDs in the three products was used to fabricate a white light-emitting diode. The white light-emitting diode shows an excellent colour rendering index up to 93.3, suggesting broad application prospects of the CDs in lighting and display fields.

## Abstract 2

Yellow-green photoluminescence carbon dots with different quantum yields were realized and used to fabricate white LEDs.

## Graphical abstract

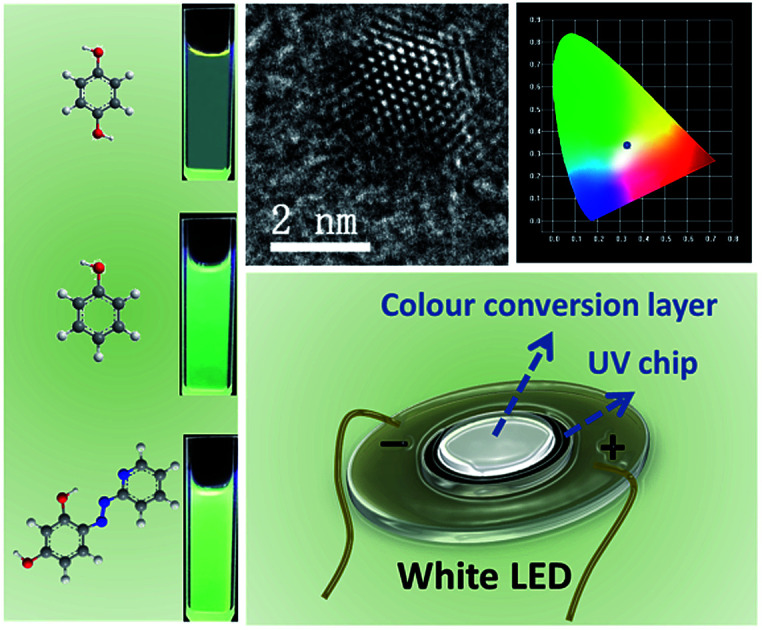

## 1. Introduction

Fluorescent carbon dots (CDs), a kind of carbon materials, have received increasing interest since they were first found in 2004.^1^ For their unique properties, such as high water solubility, tunable emission, low toxicity, and facile preparation, CDs have received much attention and show great application value in many fields including biological imaging, cancer treatment, sensors, photoelectric devices and catalysis.^2–5^ For example, light-emitting diodes using CDs to work as colour conversion layers have been reported by several groups and show a broad development prospect.^6–8^

Though various precursors and methods have been applied on the preparation of CDs, most of the products show blue-light-region emission.^9–12^ Our group achieved blue emissive CDs using citric acid as the only carbon source *via* an ammonium hydroxide modulated method, the obtained CDs show a photoluminescence quantum yield (PL QY) of 40%.^13^ However, blue emission is often interfered with by self-fluorescence of biological matrixes and common organic compounds.^14^ Therefore, it is still a burning question to develop methods for synthesizing high-efficient fluorescent CDs of emission at longer wavelength. To achieve this aim, research on regulating the fluorescence of CDs under controlled, comparable conditions are in great demand.

To date, there is no unified view upon the fluorescence mechanism of CDs.^15^ Many researchers proposed that the surface states of the CDs may be the origin of their fluorescence behaviour.^16,17^ Lin and his group ascribed the excitation-independent PL features of CDs to the surface groups/defects emission especially π systems containing C–N/C=N or C–O structure.^18^ Xiong's group agreed that the surface molecular state would be mostly responsible for the PL emission.^19^ To obtain CDs of better fluorescence properties, surface modification and heteroatom doping especially nitrogen doping that can change the surface states and improve the photoluminescence of CDs dramatically, were frequently used.^20–23^

Employing aromatic compound as carbon source in the synthetic process can make significant differences to the preparation of high quality long-wavelength-emissive CDs. For example, Jiang and co-workers reported the preparation of multicolour PL CDs, having red, green, and blue emissions by using the three different phenylenediamines isomers, through a solvothermal method.^24^ Recently, they also prepared yellow emissive CDs using 1,2,4-triaminobenzene as carbon source.^14^ Guo and co-workers synthesized yellow-green-emissive CDs (QY 6.59%) from 2-hydroxyphenylboronic acid.^25^ Yang's group realized yellow-green-emissive CDs (QY 28.6%) from *m*-aminophenol.^26^ Ding *et al.* reported full-colour light-emitting carbon dots prepared from *p*-phenylenediamine and urea through hydrothermal method.^27^

In this work, three phenol derivatives with aromatic structures, especially 4-(2-pyridylazo) resorcinol with a pyridine ring and an azo bond in its structure were chosen as carbon source to achieve CDs of high nitrogen-doped level and long-wavelength emission. Three efficient yellow-green emissive N-doped CDs of the same fluorescence peak wavelength but different PLQY were realized through a facile hydrothermal method. The CDs prepared from 4-(2-pyridylazo) resorcinol have the highest PLQY of 24.4% (in water, and 53.3% in ethanol) among the three CDs. And this is ascribed to the high nitrogen content and abundant surface functional groups of the CDs. After washing and reducing these prepared CDs which have similar surface states, the emission evolution was studied linking the surface defects and the changes of surface bonds. In addition, the best CDs in the three samples was further used as a yellow-green colour conversion layer to fabricate white emissive light-emitting diode. The prepared white emissive light-emitting diode shows bright light with a high colour rendering index up to 93.3, the results suggest great potential of CDs in the fields of lighting and display.

## 2. Experimental

### 2.1 Reagents

All chemicals were purchased from commercial supplies and are of analytical grade. Hydroquinone, phenol, 4-(2-pyridylazo) resorcinol, and transparent epoxy (JH-6800MA and JH-6800MB) used for light-emitting diode were obtained from Aladdin Biochemical Technology Co., Ltd. (Shanghai, China). Ethylenediamine, methanol, acetone and ethanol were purchased from Sinopharm Chemical Reagent Co., Ltd. (Shanghai, China). The 360 nm LED chips with a power of 1 W were provided by APT Electronics Ltd. Red-emitting phosphor (Sr,Ca)AlSiN_3_:Eu was obtained from Shenzhen Looking Long Technology Co., Ltd. All reagents were used as received without further purification. The ultrapure water with 18.2 MΩ cm^−1^ (Millipore Simplicity, USA) was used throughout the syntheses and spectroscopic measurements.

### 2.2 Equipment

Transmission electron microscopy (TEM) and high resolution TEM (HRTEM) observations were performed on a JEM-2100 field emission source transmission electron microscope operating at 200 kV. X-ray diffraction (XRD) patterns were recorded using an X-ray diffractometer (Rigaku, Japan) with Cu Kα radiation (*λ* = 1.540 Å). The 2*θ* scanning range was from 10° to 70° with a scanning speed of 0.1° s^−1^. Fourier transform infrared (FT-IR) spectra were obtained by a Nicolet 67 FT-IR spectrometer (Thermo Nicolet, America). X-ray photoelectron spectroscopy (XPS) was carried out on a Thermo Escalab-250 X-ray photoelectron spectrometer. Fluorescence emission and excitation spectra were measured using a Hitachi F-4600 fluorescence spectrophotometer. UV-vis absorption spectra were recorded by a Shimadzu UV-2550 spectrophotometer. Raman spectra were measured on Lab Ram HR Evolution of HORIBA JOBIN YVON. Time-correlated single-photon counting (TCSPC) data were obtained on an Edinburgh FL 900 photo-counting system. The optical properties of the as-fabricated light-emitting diodes were tested using an integrating sphere (Everfine Photo-E-Info Co., Ltd). Fluorescent photographs were taken under excitation of 365 nm by a ZT-5 hand-held UV lamp (Jiapeng, China).

### 2.3 Synthetic procedures

Experiments of the three CDs were conducted in parallel. The CDs can be facilely prepared through a typical synthesis process. A phenol derivative (5 mmol) and ethylenediamine (300 μL, 4.5 mmol) were dissolved adequately in 30 mL ultrapure water after a sonication (20 min), then the homogeneous solution was transferred into a 50 mL poly(tetra-fluoroethylene) (Teflon)-lined stainless steel autoclave. After being heated at 200 °C for 6 h and then cooled to room temperature naturally, the reaction product was filtered and centrifuged at 5000 rpm for 20 min to remove larger particles. The resulting supernatant was concentrated and then washed with acetone and 10% methanol/acetone two times (30 mL for each wash step). After being dried in vacuum oven at 60 °C, the three CDs were finally obtained as dark brown powders. To investigate the relationship between surface states and photoluminescence of the CDs, the washing times that after the hydrothermal synthetic process was added to six, and the obtained powders (denoted as W-CDs) were further reduced by hydrogen at 400 °C for 5 hours in a tube furnace (the product was named as F-CDs) to remove the surface functional groups as many as possible.

### 2.4 Fabrication of WLED

Typically, the yellow-green-emission CDs powder (prepared from 4-(2-pyridylazo) resorcinol, named as R-CDs) and blue-emission CDs powder (which was synthesized following our previous work,^13^ denoted as B-CDs) were dissolved in ethanol. Equal weight of transparent epoxy JH-6800MA and JH-6800MB were mixed together and then added with the red-emission (Sr,Ca)AlSiN_3_:Eu powders, R-CDs solution and B-CDs solution. The mixture was stirred sufficiently to become homogenous and then dispersed on a 360 nm LED chip. The coated LED chip was solidified in a vacuum oven at 40 °C for 60 min and thermally cured at 100 °C for 2 h.

### 2.5 Determination of QY

QY was measured according to the widely accepted procedure by using rhodamine 6G (QY = 95% in ethanol) as the standard.^14^ The QY was calculated with the following equation
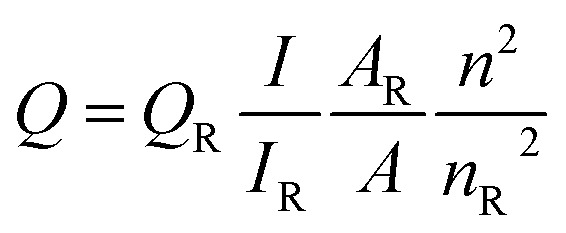
where *Q* is the QY, *I* is the measured integrated emission intensity, *A* is the optical density and *n* is the refractive index (1.33 for water and 1.36 for ethanol). The subscript R refers to the reference dye of known QY. To minimize reabsorption effects, absorption intensity was kept below 0.05 at the excitation wavelength. Each experiment was performed three times in parallel to obtain the average QY value.

## 3. Results and discussion

**Fig. 1:**
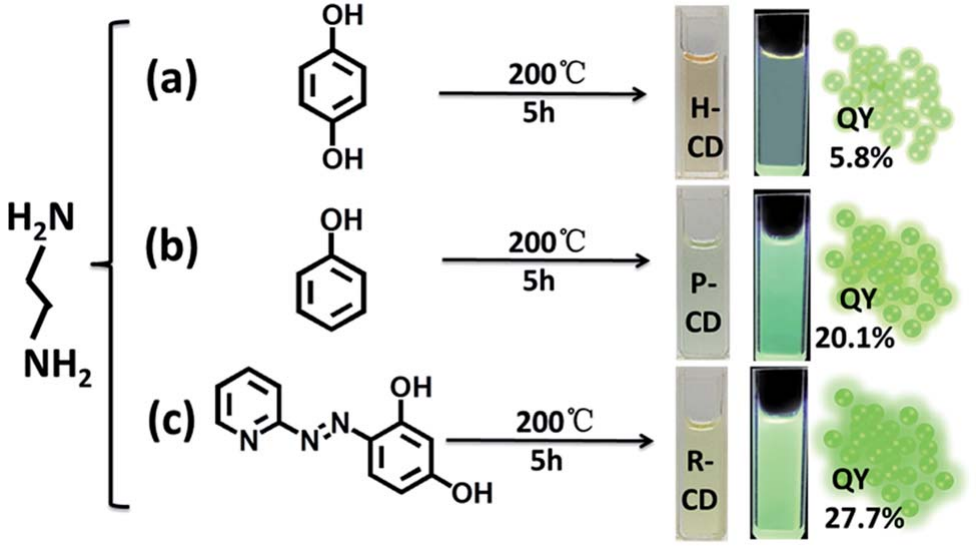
Synthesis conditions of ethylenediamine-modulated carbon dots (CDs) using three different carbon sources. (a) Reaction of hydroquinone and ethylenediamine, resulting in H-CDs, (b) reaction of phenol with ethylenediamine, producing P-CDs, (c) reaction of 4-(2-pyridylazo) resorcinol and ethylenediamine, resulting in R-CDs, (a–c) images of the water solution of the samples in day light and under UV light excitation which display yellow-green emission with PL QYs labelled on the graphs.

Ethylenediamine and three different carbon sources: hydroquinone, phenol and 4-(2-pyridylazo) resorcinol were adopted in the hydrothermal synthetic processes of the CDs, correspondingly the samples are denoted as H-CDs, P-CDs and R-CDs respectively (Fig. 1, and also refer to the Experimental section for details). The crude products were filtered and centrifuged to remove agglomerated large particles, and then washed for two times to get rid of the unreacted reagents. The resulting samples are named as raw CDs. Pink, green and yellow solutions (for H-CDs, P-CDs and R-CDs) can be observed when they are dispersed in ultrapure water. Interestingly, all of the solutions display the emission of 530 nm under single-wavelength UV irradiation (*e.g. λ* = 365 nm).

**Fig. 2:**
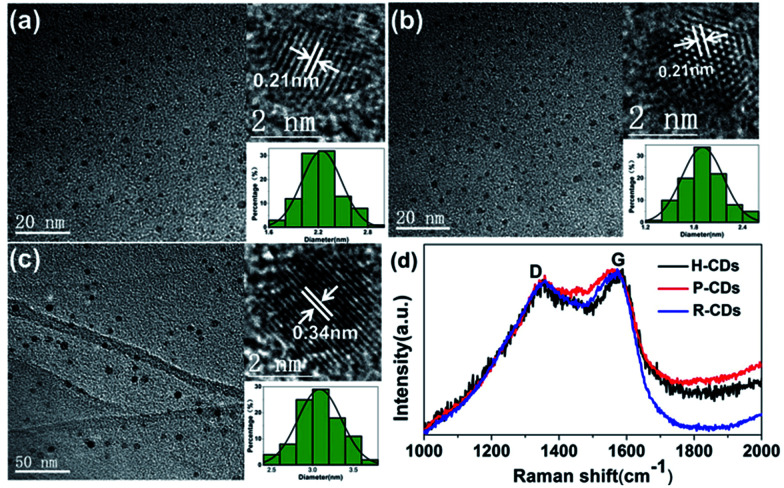
TEM and Raman tests results. TEM and HRTEM (inset) images of H-CDs (a), P-CDs (b), and R-CDs (c); Raman spectra (d) of the CDs.

Transmission electron microscopy (TEM) tests were carried out to evidence our successfully preparation of CDs. As shown in Fig. 2a–c, the CDs are well-dispersed and display uniform nanoparticles with average sizes about 2.3 nm (H-CDs), 2.05 nm (P-CDs) and 3.02 nm (R-CDs), respectively. The particle size distributions of the CDs are so different that their shared luminescence peak wavelength obviously cannot be ascribed to the same particle size. The high-resolution TEM images providing in the insets show that the CDs exhibit well-resolved lattice fringes with spacing of 0.21 nm and 0.34 nm which are corresponding to the (100) and (002) facet of graphite. The obtained Raman spectra (Fig. 2d) agree well with the TEM results. All of the three CDs have two peaks at about 1578 cm^−1^ (G band) and 1352 cm^−1^ (D band) under background of fluorescence, indicating sp^2^ carbon networks and the disorder or defects in the surface structure of carbon dots. Obvious D and G bands detected in the test evidence the good crystallinity of the samples. The XRD patterns of the CDs (Fig. 6d) all display broad diffraction peaks centred at 24°, corresponding to an interlayer distance of 0.37 nm close to graphite (0.34 nm), the increase is ascribed to abundant surface groups of the CDs.^28^

**Fig. 3:**
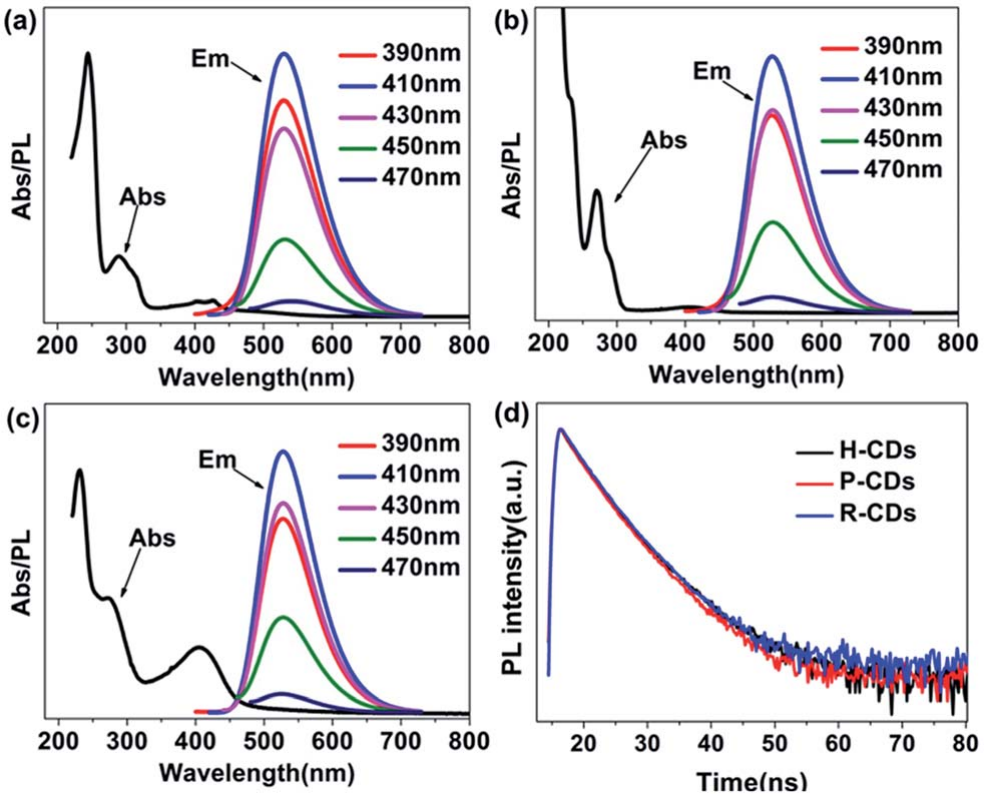
Absorption and photoluminescence (PL) emission spectra of raw CDs: H-CDs (a), P-CDs (b) and R-CDs (c) under excitation light of different wavelengths (the inset legends), time-resolved photoluminescence decay curves (d) of the CDs.

Subsequently, the optical properties of the as-prepared CDs were studied in water solution. Firstly, the ultraviolet-visible absorption spectra (Fig. 3a–c) clearly show that the three CDs have analogous characteristics. All of them have absorption bands at 230 nm, 270 nm and 405 nm, which are assigned to π–π* (aromatic C–C), π–π* (aromatic C=C) and complicated surface states, respectively.^14,19,20^ Different from many other reported CDs, the PL emission peaks of our samples do not shift under different wavelength excitations.^17^ The highest emission intensities can be observed when the excitation wavelength is set at 405 nm, revealing that the surface states are responsible for the yellow-green emission.^14^ What is more, the time-resolved photoluminescence decay measurements (Fig. 3d) of the samples were monitored at 370 nm. The calculated average PL decay lifetime of the H-CDs, P-CDs and R-CDs are 3.75 ns, 3.73 ns and 3.84 ns respectively. The similar plots are further evidences that the three samples have identical absorption structures and luminescent centres. The PLQYs were determined to be 5.8%, 20.1% and 24.4% for H-CDs, P-CDs and R-CDs, respectively (see Experimental section). When these CDs are dispersed in ethanol, all of the solutions show photoluminescence peaks locating at 490 nm which are also independent of excitation wavelength (Fig. S1†). This solvent-dependent fluorescence could be caused by a dipole induced surface electronic state change which has been reported by Wang *et al.*^29^ Being measured in ethanol solution, the PLQYs reached 27.7%, 49.2% and 53.3% for H-CDs, P-CDs and R-CDs.

**Fig. 4:**
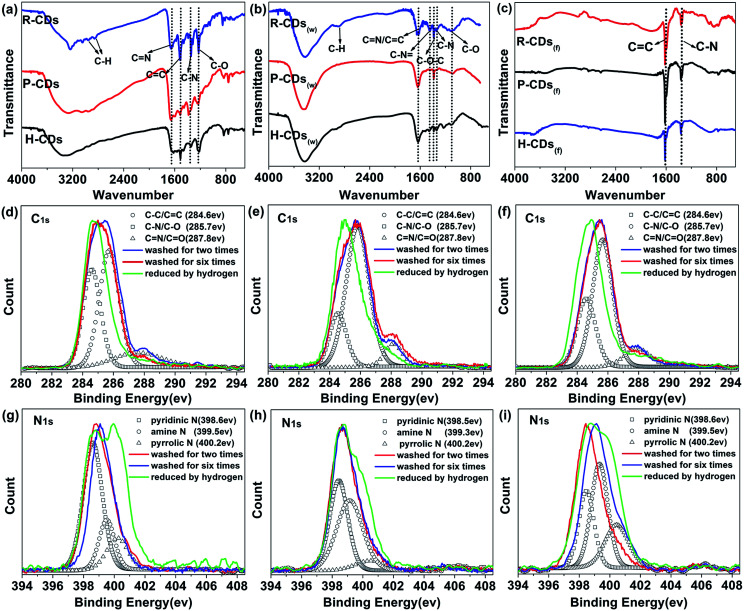
Characterization of the surface composition and chemical state of the CDs. (a–c) FT-IR characterization of the CDs washed for two times, six times and further reduced by hydrogen, respectively; (d–i) XPS survey; high-resolution XPS were de-convoluted following the literature: (d and g) for H-CDs, (e and h) for P-CDs and (f and i) for R-CDs.

To identify the surface compositions and chemical states of the CDs, Fourier transform infrared spectroscopy (FT-IR) and X-ray photoelectron spectroscopy (XPS) were carried out (Fig. 4). As shown in Fig. 4a, the three raw CDs exhibit similar FT-IR spectra, indicating the existences of similar chemical compositions. The absorbing at 1850–3000 cm^−1^ are attributed to –OH groups in strong hydrogen bonding while absorption bands at 3050–3650 cm^−1^ are assigned to *ν*(O–H) and *ν*(N–H).^30^ The peaks between 2840 and 2960 cm^−1^ are arising from C–H stretching vibrations of methyl/methylene.^18,30^ Strong stretching vibrations of C=N (1643 cm^−1^), C=C (1520 cm^−1^), C–N (1348 cm^−1^) and C–O (1200 cm^−1^) are observed from each sample, implying the formation of poly-aromatic structures in the CDs during the reaction process.^31^ A comparison of the FT-IR spectra of the three samples reveals two important observations. One is that the vibration bands at approximately 3240 cm^−1^ and 3050 cm^−1^ gradually change from integrated to discrete from H-CDs to P-CDs to R-CDs. The broad absorption bands indicate that the amino (–NH_2_) and hydroxyl (–OH) groups on the surface of the CDs belong to multiple structures, leading to higher hydrophilicity and polarity of the samples.^27^ Another is that the R-CDs exhibits stronger signals of the C=N, C=C and C–N stretching vibrations when compared with P-CDs and H-CDs, implying that the increase of surface functional groups and the enhancement of N-doping degree would benefit the PLQY enhancement of the CDs.

The FT-IR assignments were confirmed by XPS analysis. The XPS results illustrate that the CDs mainly contain three elements: C, N and O. In the high-resolution spectra (Fig. 4), the C_1s_ band can be de-convoluted into three peaks (*i.e.*, 284.6 eV (C–C/C=C), 285.7 eV (C–N/C–O), and 287.8 eV (C=N/C=O)). And the N_1s_ band contains three peaks at 398.7 eV, 399.1 eV and 400.2 eV, representing pyridinic N, amino N and pyrrolic N respectively.^27,32^ The atomic ratio between nitrogen and carbon increases from 0.22 to 0.29 as the N content increases from 15.45 to 22.6%, from H-CDs to P-CDs to R-CDs (Table S1†). A positive correlation between the degree of N doping and the fluorescence properties of the CDs can be deduced.

When compare the three carbon sources, 4-(2-pyridylazo) resorcinol shows great differences from the others. The pyridine ring and an azo bond in its chemical structure allow a higher nitrogen content and formation of more heterocycle chemical structures in the corresponding product, thus enable the R-CDs to be the best in the three CDs.

**Fig. 5:**
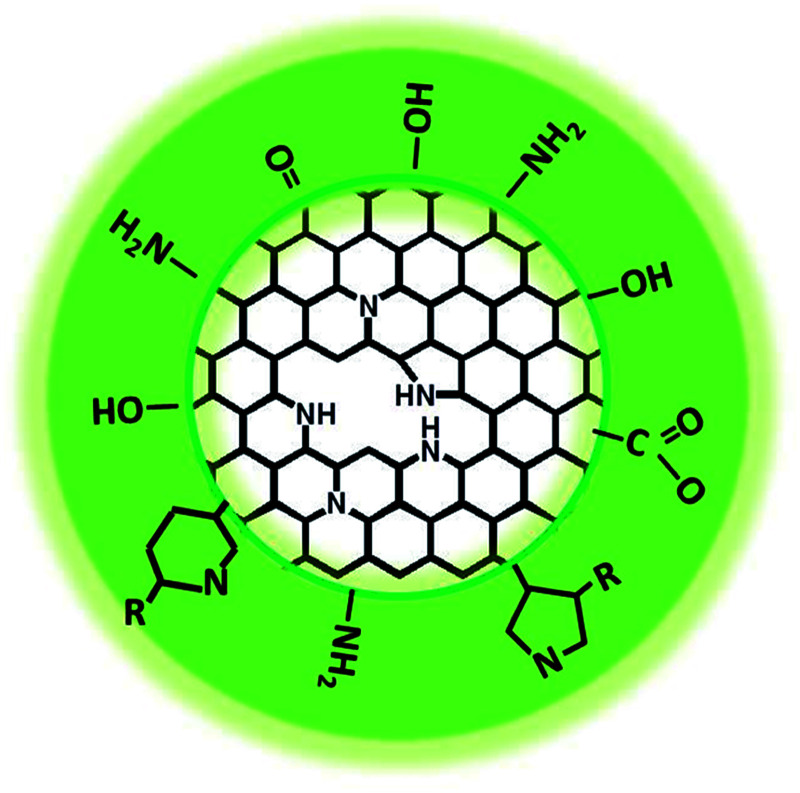
Structural model of the as-prepared CDs.

Based on the experimental results and characterization data including the TEM, Raman, FT-IR, and XPS results, the CDs could be tentatively considered to be composed of π-conjugated domains in the carbon cores and amorphous regions on their edges, with abundant amide, hydroxyl and poly-aromatic groups on the surface (Fig. 5).

**Fig. 6:**
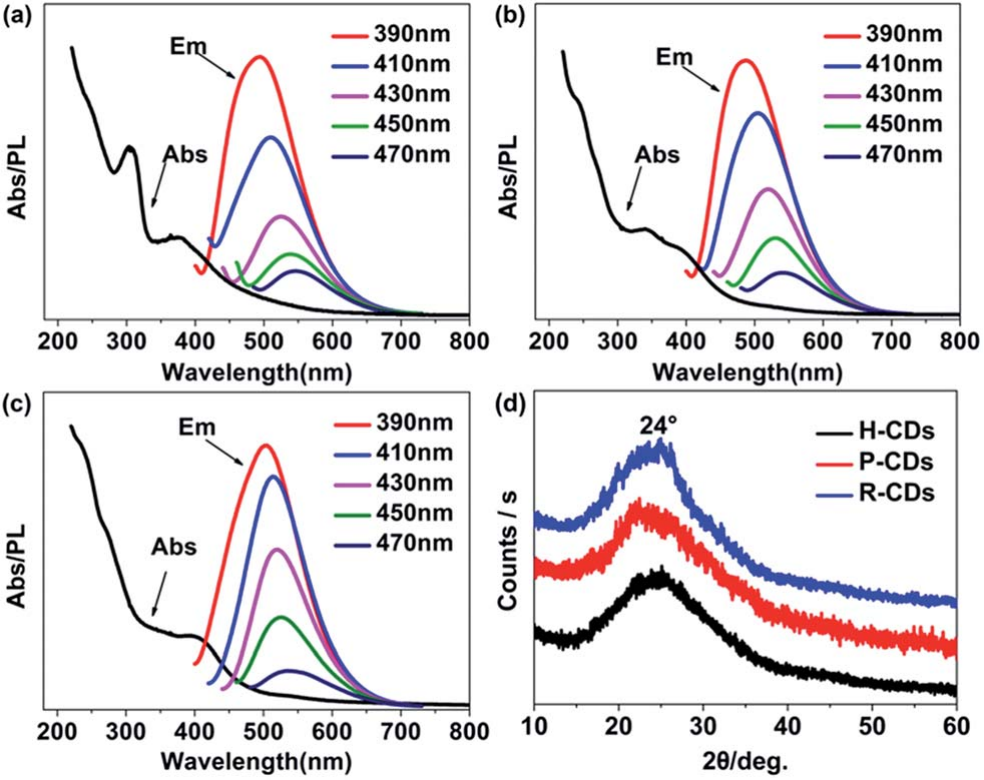
Absorption and photoluminescence (PL) emission spectra of the CDs washed for six times: H-CDs_(w)_ (a), P-CDs_(w)_ (b) and R-CDs_(w)_ (c) under excitation light of different wavelength (the inset legends); XRD patterns of the three CDs (d); CD_(w)_ is for the six-times-washed CDs.

To figure out how the surface groups and surface defects affect the luminescence properties of the CDs, the raw CDs were washed (resulting products were denoted as W-CDs) and further reduced by hydrogen (resulting products were named F-CDs) to remove the surface groups as many as possible. Not surprisingly, the W-CDs show obvious excitation-dependent fluorescence with a sharply decline of PLQY when compared to the raw CDs. The absorption and PL emission spectra of the W-CDs were tested (Fig. 6). As altering the excitation wavelengths from 390 to 470 nm, the emission peaks of the W-CDs shift from 480 to 530 nm. Moreover, no fluorescence could be observed when the F-CDs were excited. Above results prove that the fluorescence in the CDs is originated from abundant surface functional groups on the surface, rather than the carbon core. Emission evolution of the CDs was studied linking the surface defects and the changes of surface bonds.

The FT-IR characterization results of the W-CDs and F-CDs exhibit great differences from the raw CDs'. As shown in Fig. 4b and c, the surface groups of CDs decreased significantly after careful wash, and only C=C and C–N survived after hydrogen reduction. Conclusions can be drawn that the surface functional groups do affect the PL of CDs, especially for the excitation-independent PL. Researchers usually ascribe the excitation-dependent fluorescence to the different photon absorption of the CDs, *i.e.* wide distribution of differently sized dots, surface chemistry or emissive traps.^33^ Our researches agree with the view that different surface states lead to different emission wavelengths, when the CDs are fully passivated by a certain kind of functional groups that generate fluorescence, excitation-independent PL is to appear.^9,16^

According to Fig. 4b and c, the blue-green emission of the W-CDs vanished with the elimination of the O–H (3425 cm^−1^), N–H (3200 cm^−1^), C=N (1643 cm^−1^), C–N= and C–O bonds.^34^ The FT-IR results of W-CDs matches well with our previous work's,^13^ in which CDs with bright blue fluorescence were developed. Compare Fig. 4b with 4a, the signals from N–H, C=N, C=C and C–N bonds show obviously decrease, corresponding to the weakening of yellow-green emission after the wash process.

From the XPS spectra (Fig. S2†), the nitrogen content in the CDs decreased after the wash and the reduction processes (Table S1†), further prove that the existing of nitrogen on the surface is conducive to the fluorescence of the CDs. The more nitrogen the CDs have, the higher PLQY they possess. It's worth noting that different content, ratios or species of doped N could affect PL properties of N-doped CDs.^10,35^ The high-resolution XPS patterns of W-CDs and F-CDs (C_1s_ and N_1s_) (Fig. 4d–i) reveal that the C=N/C=O bonds, amine N and pyridinic N reduced significantly after the wash process, while the C–N/C–O, C=N/C=O and pyridinic N decreased drastically after the hydrogen reduction. Through carefully comparison and analysis, we can speculate that the amidogen and heterocycle chemical structures play a vital role in the radiative transition of long wavelength emission, while C–N or oxygen-related groups like –OH and C–O trigger the blue-green emission.

Heteroatom doping, which can form defect energy level, is also considered to have a role in modifying the performance of CDs.^36,37^ Pyridinic N, pyrrolic N and even oxygen-related structures can affect the large conjugated structures on the edge of graphitic framework and generate more surface defect states which dominate surface-state-related PL of the CDs.^38^ After wash and hydrogen reduction, the obtained F-CDs could be loosely thought of as carbon cores with few surface groups which can be ignored. Compare F-CDs with W-CDs and raw CDs (Fig. 4g–i), as the cleaning processes goes on, the signal of pyrrolic N enhances gradually, showing that pyridinic N is easier to remove than pyrrolic N, which means more pyridinic N located at the surface while pyrrolic N doped in the carbon framework. Due to the special five-membered ring, pyrrolic N could cause great damage to the integrity of graphitic framework in the core of carbon dots.^10^ According to the well-resolved lattice fringes of the CDs in the HRTEM results, it could be inferred that the pyrrolic N are distributed on the edge of graphitic framework rather than in the core.

**Fig. 7:**
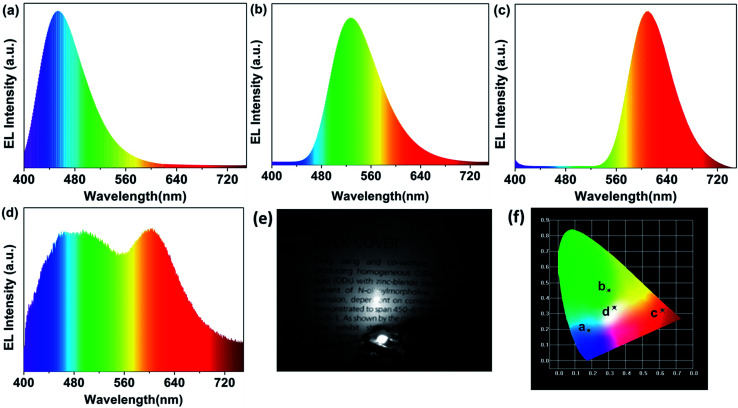
(a–d) Electroluminescence spectrum of the blue, yellow-green, red and white LED; (e) the photograph of the WLED in the dark; (f) their corresponding International Commission on Illumination (CIE) colour coordinates.

Serving as a yellow-green colour conversion layer, the R-CDs were used to fabricate a white light-emitting diode (WLED). Having a CIE colour coordinate of (0.3316, 0.3373), a CRI up to 93.3 and a colour temperature (*T*_c_) of 5538 K at 100 mA, the as-prepared WLED exhibits a wide emission in the electroluminescence (EL) spectrum (Fig. 7d). Herein, the R-CDs, blue-emission CDs (reported in our previous work^13^) and red emission (Sr,Ca)AlSiN_3_:Eu powders were used as colour conversion layers and a 360 nm UV chip as excitation light source (Fig. 7e). The UV light emitted from the chip excites the mixture of fluorescent materials, leading the as-prepared LED to emit bright white light. The LED only using the three ingredients as conversion layer was also fabricated to investigate the role they play in the property of the WLED. Blue LED by blue-emission CDs (*λ*_em_ = 460 nm), yellow-green by R-CDs and red LED by (Sr,Ca)AlSiN_3_:Eu powders (*λ*_em_ = 610 nm) are shown in Fig. 7a–c. The CIE coordinates of the blue, yellow-green and red LED are (0.18, 0.19), (0.30, 0.44) and (0.62, 0.33), respectively (shown in Fig. 7f). The excellent performance of the WLED with a high colour rendering index and a low colour temperature indicates that the CDs with excellent optical properties will have broad application prospect in optoelectronic fields.

## 4. Conclusions

In summary, three kinds of yellow-green emissive N-doped carbon dots with excellent crystallization and the same fluorescence peak wavelength were synthesized through a facile hydrothermal of phenol derivatives. The as-prepared CDs exhibit not only excited-independent emission but also high photoluminescence quantum yields. Abundant surface functional groups and high N-doping degree are responsible for their excellent performances. The CDs which shows the highest nitrogen content also has the highest QY up to 24.4% in water and 53.3% in ethanol. By washing and reducing the raw CDs which have similar surface states, emission evolution of the CDs was analysed linking the surface bonds and the carbon frame work with the PL properties. The results indicated that the surface states play important roles in the fluorescence of CDs. Furthermore, a WLEDs has been fabricated with the R-CDs, B-CDs (reported in our previous work) and red emission (Sr,Ca)AlSiN_3_:Eu powders excited by 360 nm UV chips. The as-fabricated WLED exhibits bright light with an attractive CRI up to 93.3, a *T*_c_ of 5538 K at 100 mA, suggesting that the CDs are promising conversion materials in lighting and display field.

## Conflicts of interest

There are no conflicts to declare.

## Supplementary Material

RA-008-C7RA12522D-s001

## References

[cit1] Xu X., Ray R., Gu Y., Ploehn H. J., Gearheart L., Raker K., Scrivens W. A. (2004). J. Am. Chem. Soc..

[cit2] Du Y., Guo S. (2016). Nanoscale.

[cit3] Campos B. B., Contreras-Cáceres R., Bandosz T. J., Jiménez-Jiménez J., Rodríguez-Castellón E., Esteves da Silva J. C. G., Algarra M. (2016). Carbon.

[cit4] Peng Z., Miyanji E. H., Zhou Y., Pardo J., Hettiarachchi S. D., Li S., Blackwelder P. L., Skromne I., Leblanc R. M. (2017). Nanoscale.

[cit5] Wang Z., Long P., Feng Y., Qin C., Feng W. (2017). RSC Adv..

[cit6] Zhang F., Feng X., Zhang Y., Yan L., Yang Y., Liu X. (2016). Nanoscale.

[cit7] He J., He Y., Chen Y., Lei B., Zhang H., Zhuang J., Zheng M., Liu Y. (2016). RSC Adv..

[cit8] Wang Y., Wang K., Han Z., Yin Z., Zhou C., Du F., Zhou S., Chen P., Xie Z. (2017). J. Mater. Chem. C.

[cit9] Wen Z.-H., Yin X.-B. (2016). RSC Adv..

[cit10] Hu R., Li L., Jin W. J. (2017). Carbon.

[cit11] Liu Y., Zhou L., Li Y., Deng R., Zhang H. (2017). Nanoscale.

[cit12] Zhu S., Meng Q., Wang L., Zhang J., Song Y., Jin H., Zhang K., Sun H., Wang H., Yang B. (2013). Angew. Chem., Int. Ed..

[cit13] Wang S., Zhu Z., Chang Y., Wang H., Yuan N., Li G., Yu D., Jiang Y. (2016). Nanotechnology.

[cit14] Jiang K., Sun S., Zhang L., Wang Y., Cai C., Lin H. (2015). ACS Appl. Mater. Interfaces.

[cit15] Sk M. A., Ananthanarayanan A., Huang L., Lim K. H., Chen P. (2014). J. Mater. Chem. C.

[cit16] Nguyen V., Si J., Yan L., Hou X. (2016). Carbon.

[cit17] Gan Z., Xu H., Hao Y. (2016). Nanoscale.

[cit18] Sun S., Zhang L., Jiang K., Wu A., Lin H. (2016). Chem. Mater..

[cit19] Chen J., Wei J. S., Zhang P., Niu X. Q., Zhao W., Zhu Z. Y., Ding H., Xiong H. M. (2017). ACS Appl. Mater. Interfaces.

[cit20] Zhu S., Song Y., Zhao X., Shao J., Zhang J., Yang B. (2015). Nano Res..

[cit21] Zhang J., Yuan Y., Liang G., Yu S. H. (2015). Adv. Sci..

[cit22] Zhu Z., Wang S., Chang Y., Yu D., Jiang Y. (2016). Carbon.

[cit23] Zheng H., Wang Q., Long Y., Zhang H., Huang X., Zhu R. (2011). Chem. Commun..

[cit24] Jiang K., Sun S., Zhang L., Lu Y., Wu A., Cai C., Lin H. (2015). Angew. Chem..

[cit25] Guo Y., Chen Y., Cao F., Wang L., Wang Z., Leng Y. (2017). RSC Adv..

[cit26] Wang Q., Zhang S., Zhong Y., Yang X. F., Li Z., Li H. (2017). Anal. Chem..

[cit27] Ding H., Yu S.-B., Wei J.-S., Xiong H.-M. (2016). ACS Nano.

[cit28] Strauss V., Margraf J. T., Dolle C., Butz B., Nacken T. J., Walter J., Bauer W., Peukert W., Spiecker E., Clark T., Guldi D. M. (2014). J. Am. Chem. Soc..

[cit29] Wang H., Sun C., Chen X., Zhang Y., Colvin V. L., Rice Q., Seo J., Feng S., Wang S., Yu W. W. (2017). Nanoscale.

[cit30] Qu S., Zhou D., Li D., Ji W., Jing P., Han D., Liu L., Zeng H., Shen D. (2016). Adv. Mater..

[cit31] Qu D., Zheng M., Zhang L., Zhao H., Xie Z., Jing X., Haddad R. E., Fan H., Sun Z. (2014). Sci. Rep..

[cit32] Schneider J., Reckmeier C. J., Xiong Y., von Seckendorff M., Susha A. S., Kasák P., Rogach A. L. (2017). J. Mater. Chem. C.

[cit33] Wang Y., Li Y., Yan Y., Xu J., Guan B., Wang Q., Li J., Yu J. (2013). Chem. Commun..

[cit34] Tian R., Hu S., Wu L., Chang Q., Yang J., Liu J. (2014). Appl. Surf. Sci..

[cit35] Qian Z., Ma J., Shan X., Shao L., Zhou J., Chen J., Feng H. (2013). RSC Adv..

[cit36] Sarkar S., Sudolská M., Dubecký M., Reckmeier C. J., Rogach A. L., Zbořil R., Otyepka M. (2016). J. Phys. Chem. C.

[cit37] Ding H., Wei J. S., Xiong H. M. (2014). Nanoscale.

[cit38] Qian Z., Ma J., Shan X., Feng H., Shao L., Chen J. (2014). Chemistry.

