# Supplementary material for: N-doped carbon dots from phenol derivatives for excellent colour rendering WLEDs
Source: RSC Adv. 2018 Jan 26;8(9):4850–6. doi: 10.1039/c7ra12522d (PMC9077792; doi:10.1039/c7ra12522d)
Supplement: RA-008-C7RA12522D-s001 [file RA-008-C7RA12522D-s001.pdf]

Electronic Supplementary Material (ESI) for RSC Advances.
This journal is © The Royal Society of Chemistry 2018

Supporting Information

N-Doped carbon dots from phenol derivatives for excellent colour rendering WLEDs

Qian Liu [a], Danting Li [a], Zhifeng Zhu [a], Shimeng Yu [a], Yan Zhang [a], Dabin Yu [b],, and Yang Jiang [a],**

[a] School of Materials Science and Engineering, Hefei University of Technology, Hefei 230009, P. R. China; cathyliuq@163.com(Q.L.); wlkd1106t@163.com(D.L.); zhuzhifeng@hfut.edu.cn(Z.Z.) ; yushimenglxl@163.com (S.Y.); yanzi813@163.com (Y.Z.)

[b] State Key Laboratory of Pulsed Power Laser Technology, National University of Defense Technology, Hefei 230037, P. R. China;

* Corresponding authors: apjiang@hfut.edu.cn. (Y. J.), dbyu@ustc.edu(D. Y.).

Figures

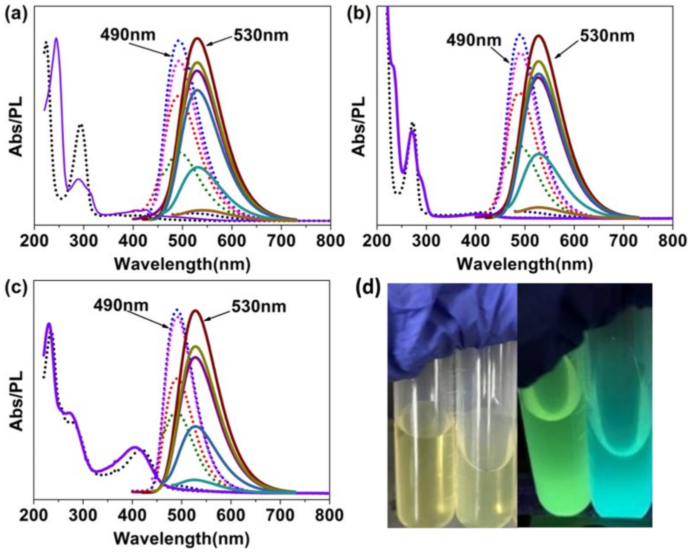

Fig. S1 Absorption and photoluminescence emission spectra of H-CDs(a), P-CDs(b) and R-CDs(c) in solvent of water(solid line) and ethanol(dash line) under excitation light of different wavelengths (the inset legends); True color of CDs in daylight and under UV light(365nm)

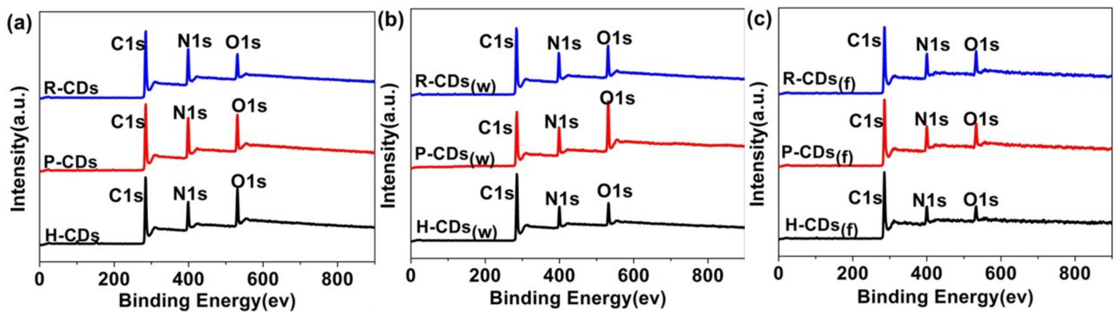

Fig. S2 XPS spectra of the raw CDs(a), W-CDs(b) and F-CDs(c)

Tables

Table S1 Percentages of N atoms in CDs roughly estimated by the results of XPS

	H-CDs	P-CDs	R-CDs
Raw CDs	15.45%	21.4%	22.6%
W-CDs	12.8%	16.4%	16.9%
F-CDs	11.4%	15.3%	15.8%
